# Risk-Reducing Options for High-Grade Serous Gynecologic Malignancy in BRCA1/2

**DOI:** 10.3390/curroncol29030172

**Published:** 2022-03-21

**Authors:** Lauren Clarfield, Laura Diamond, Michelle Jacobson

**Affiliations:** 1 Temerty Faculty of Medicine, University of Toronto, Toronto, ON M5S 1A8, Canada; lauren.clarfield@mail.utoronto.ca (L.C.); laura.diamond@mail.utoronto.ca (L.D.); 2 Department of Obstetrics and Gynecology, University of Toronto, Toronto, ON M5G 1E2, Canada; 3 Department of Obstetrics and Gynecology, Mount Sinai Hospital, Toronto, ON M5T 2Z5, Canada; 4 Department of Obstetrics and Gynecology, Women’s College Hospital, Toronto, ON M5S 1B2, Canada

**Keywords:** ovarian neoplasms, genes, BRCA 2, genes, BRCA 1, endometrial neoplasms, contraceptives, oral, hysterectomy, salpingo-oophorectomy, breast neoplasms, genetic predisposition to disease, germ-line mutation

## Abstract

Ovarian cancer (OC) is the leading cause of death among women with gynecologic malignancy. Breast Cancer Susceptibility Gene 1 (BRCA 1) and Breast Cancer Susceptibility Gene 2 (BRCA 2) germline mutations confer an estimated 20 to 40 times increased risk of OC when compared to the general population. The majority of BRCA-associated OC is identified in the late stage, and no effective screening method has been proven to reduce mortality. Several pharmacologic and surgical options exist for risk-reduction of gynecologic malignancy in BRCA 1/2 mutation carriers. This review summarizes up-to-date research on pharmacologic risk-reducing interventions, including the oral contraceptive pill, acetylsalicylic acid/nonsteroidal anti inflammatory drugs (ASA/NSAID) therapy, and denosumab, and surgical risk-reducing interventions, including risk-reducing bilateral salpingo-oophorectomy, salpingectomy with delayed oophorectomy, and hysterectomy at the time of risk-reducing bilateral salpingo-oophorectomy.

## 1. Introduction

Ovarian cancer (OC) is the leading cause of death among women with gynecologic malignancy in Canada, responsible for an estimated 1,950 deaths annually [1]. Although the majority of OC is sporadic, approximately one-quarter of affected women have a hereditary predisposition to this disease [2,3]. Pathogenic variation in the Breast Cancer Susceptibility Genes 1 and 2 (BRCA 1/2) is the strongest genetic risk factor for OC, responsible for 15% of all cases [2,3]. Although the risk of OC in the general population is less than 2%, patients with germline mutations in BRCA 1/2 face a cumulative lifetime risk of OC of 36–53% and 11–25%, respectively [4,5]. 

BRCA 1/2 are tumor-suppressor genes which have been implicated in several fundamental cellular processes, including DNA repair, cell-cycle control, transcriptional regulation, protein ubiquitylation and chromatin remodeling [6]. Specifically, DNA repair through homologous recombination repair is thought to be the primary method of tumor suppression [7]. According to the two-hit paradigm of tumor development, patients with germline mutations in BRCA 1/2 are susceptible to somatic mutations in the corresponding BRCA allele, resulting in an accumulation of chromosomal abnormalities and eventual tumorigenesis [7]. 

BRCA-associated OC has distinct clinicopathological features. In contrast to OC in the general population, BRCA-associated OC is diagnosed at an earlier median age, with an average diagnosis at 54 and 59.5 years for BRCA 1 and BRCA 2, respectively, compared to 63 years among the general population [4]. The majority of BRCA-related OC is high-grade serous carcinoma (HGSC) (67%) and endometrioid carcinoma (12%) [8]. 

Emerging evidence in the last decade suggests that BRCA-associated OC predominantly arises from fallopian tube epithelium, and spreads to the ovary secondarily [9]. For instance, the predominant histopathological subtypes resemble epithelia of the fallopian tube, as opposed to cells in the ovarian surface epithelium. In addition, pathology review of specimens from prophylactic salpingo-oophorectomy in asymptomatic BRCA mutation carriers revealed a high frequency of early carcinomas localized to the distal fallopian tube [10,11,12,13]. 

To date, no effective screening methods have been identified for OC, and the vast majority of BRCA-associated OC is identified when it has already spread to peritoneal surfaces in stage III/IV [14,15,16]. Despite promising preliminary research reviewing lavage of the uterine cavity to detect circulating tumor cells, circulating cell-free DNA, small noncoding RNAs, and tumor-educated platelets [16], risk-reducing interventions remain vital for reducing mortality among BRCA 1/2 mutation carrier patients. This review summarizes the current pharmacological and surgical risk-reducing interventions for gynecologic malignancy prophylaxis in BRCA 1/2 mutation carriers.

## 2. Discussion

### 2.1. Pharmacologic Prophylaxis

#### 2.1.1. Chemoprophylaxis with Oral Contraceptive Pill

The oral contraceptive pill (OCP) has been found to reduce the risk of OC in the general population by 40–50% [17,18,19,20,21,22]. Given that incessant ovulation is a well-known risk factor for OC, OCP is thought to reduce the risk of OC through inhibition of ovulation by negative regulation in the hypothalamus–pituitary–gonadal axis. Incessant ovulation has likewise been demonstrated as a risk factor for BRCA-associated OC. One analysis demonstrated a 45% risk-reduction for developing OC among BRCA-carrier women who had the lowest-versus-highest quartile of ovulatory cycles (OR = 0.55; 95% CI 0.41–0.75 *p* = 0.0001) [23]. Accordingly, OCP has been demonstrated to be similarly protective for OC in BRCA 1/2 mutation carriers [24,25,26].

Chemoprophylaxis with OCP in BRCA mutation carriers is complicated by heterogenous data regarding compounded increased risk of breast cancer in this high-risk group [18,27]. Huber et al. (2020) published a large systematic review highlighting the largely conflicting evidence on this association [27]. Two meta-analyses by Cibula et al. (2010) and Cibula et al. (2011) identify a mild-to-moderate increased risk of breast cancer in BRCA 1/2 mutation carriers with OCP [18,28]. Specifically, Cibula et al. (2010) identified an increased risk when the duration of OC use was at least 4 years before the first full-term pregnancy, with hazard ratios of 1.49; 95% CI 1.05–2.11, and 2.58; 95% CI 1.21–5.49 for BRCA 1 and 2 mutation carriers, respectively [28]. One study only identified a significant association for patients who started OCP before 20 years of age [29]. Conversely, several meta-analyses included in Huber et al.’s (2020) review, including those by Cibula et al. (2011), Moorman et al. (2013), and Park et al. (2017), found no increased risk of breast cancer with OCP use for BRCA 1/2 mutation carriers [18,30,31].

Newer formulations of OCP use lower doses of estrogen and progestins to decrease unwanted side-effects associated with higher doses of these hormones [32]. One meta-analysis in the general population did not identify a change in the OCP-related OC risk with lower-dose formulations [33], however, evidence from Iodice (2010) suggests that lower-dose OCPs may confer lower breast cancer risk. Specifically, they only identified an OCP-related increased risk of breast cancer with OCP formulated before 1975, compared with after, with a relative risk ratio of 1.47; 95% CI 1.06–2.04 vs. 1.17; 95% CI 0.74–1.86 [24]. 

The first prospective study on OCP and breast cancer risk in BRCA 1/2 mutation carriers was published in 2021 by Schrijver et al. (2021) [25]. Among the 6030 BRCA 1 and 3809 BRCA 2 mutation carriers included in the study, there was no increased OCP-related breast cancer risk for BRCA 1 carriers (HR = 1.08; 95% CI 0.75–1.5), but there was increased OCP-related breast cancer risk for BRCA 2 carriers (HR = 1.75; 95% CI 1.03–2.9) [25].

Overall, data on OCP-related breast cancer risk in BRCA 1/2 carriers remains limited by largely observational studies with small study populations—especially for BRCA 2 mutation carriers [27]. With informed consent regarding possibly increased breast cancer risk, the OCP can be used in BRCA 1/2 mutation carriers, serving a dual purpose as chemoprophylaxis for OC and contraception [27]. That said, there is insufficient evidence at present to recommend OCP for ovarian cancer prophylaxis in women who do not require contraception. 

#### 2.1.2. Non-OCP Hormonal Contraception 

The OCP is thought to decrease OC risk through the inhibition and suppression of pituitary gonadotropins [34]. Theoretically, then, other hormonal contraception that suppresses this axis should be similarly protective [35]. At present, data characterizing OC protection with non-OCP contraceptives, including injections, subdermal implants, and hormonal intrauterine devices, is limited mostly to the general population.

Retrospective and case–control data identify contraceptive implants as protective against OC in the general population, with a pooled odds ratio from seven case–control studies of 0.65; 95% CI 0.50–0.85 [36,37,38]. Conversely, one cohort study identified an increased risk of OC with DMPA shots compared to never users of hormonal contraceptive (RR = 6.56; 95% CI 2.11–20.40) [39].

Nexplanon, a subdermal contraceptive implant, was approved for use for contraception in Canada in 2020. A single study identified protective effects for this subdermal contraceptive, with a relative risk of 0.51; 95% CI 0.07–3.64, however, the study did not control for any history of prior contraceptive use [39].

Finally, two meta-analyses quoted protective effects for two hormonal intrauterine devices with odds ratios of 0.68 (95% CI 0.62–0.75) and 0.67 (95% CI 0.60–0.74) [40,41]. 

In summary, non-OCP hormonal contraception may confer OC protective effects; however, further research is needed, specifically in BRCA 1/2 carriers, to identify both OC and breast cancer risk associated with these contraceptive methods. 

#### 2.1.3. Denosumab 

RANK signal proteins, including RANK, RANKL, and osteoprotegerin, are expressed in OC tumor tissue, compared to non-malignant controls, with increased RANKL expression in BRCA 1/2-mutated tumors [42,43]. Denosumab, a monoclonal RANKL antibody, has been demonstrated to exert tumor-suppressive effects in mice and humans [42]. Denosumab is a largely well-tolerated drug, which is currently approved to treat osteoporosis and to prevent skeletal damage caused by bone metastasis. Denosumab’s role in the prevention of OC and breast cancer in the BRCA 1/2 carrier population is currently being investigated [44,45].

#### 2.1.4. Acetylsalicylic Acid and Non-Steroidal Anti-Inflammatory Drugs

Ovulation is classified as an inflammatory process characterized by the scheduled release of inflammatory mediators including cytokines, chemokines, prostaglandins, and steroid hormones, in response to a midcycle luteinizing hormone (LH) surge [46]. Immune cells, including neutrophils, monocytes, macrophages and mast cells, ultimately migrate to the follicle, initiating local apoptosis and eventual oocyte release. In keeping with incessant ovulation as a risk factor for OC, inflammation is known to play a role in ovarian carcinogenesis [47]. Accordingly, anti-inflammatory drugs including acetylsalicylic acid/nonsteroidal anti-inflammatory drugs (ASA/NSAIDs) have been considered for OC prophylaxis. Several studies have demonstrated a decreased risk of OC in the general population with the ever use of ASA for severe menstrual pain compared with never users (OR, 0.41; 95% CI, 0.18–0.94) [47], continuous long-term use of low-dose ASA (0.56; 95% CI 0.32–0.97) [48], and ever use of ASA (OR = 0.91; 95% CI 0.84 to 0.99) [49]. However, the current evidence for ASA and NSAIDs for OC prophylaxis is contradictory, observational, and limited by a small number of outcomes [48]. At present, there is insufficient evidence to recommend prophylactic ASA or NSAID use in BRCA 1/2 mutation carriers; however, a trial evaluating ASA and NSAID use for OC chemoprophylaxis in this population is ongoing [50].

### 2.2. Surgical Prophylaxis

#### 2.2.1. Bilateral Salpingo-Oophorectomy

Risk-reducing bilateral salpingo-oophorectomy (RRBSO) is unequivocally the gold standard for ovarian cancer prevention in the BRCA 1/2 mutation population, quoted in a 2018 Cochrane review to reduce overall mortality by 68% and HGSC-associated mortality by 94%, respectively [51]. Timing for RRBSO balances the risks of premature menopause with HGSC risk, which manifests slightly later in BRCA 2 mutation carriers compared with BRCA 1 [20,52]. Accordingly, RRBSO is recommended by the National Comprehensive Cancer Network, the Society of Gynecology Oncology, the Society of Obstetricians and Gynecologists of Canada, and the Royal College of Obstetricians and Gynecologists, for use between 35 and 40 years of age for BRCA 1 mutation carriers, and between 40 and 45 years of age for BRCA 2 carriers [53,54,55,56]. In addition to OC, RRBSO is associated with a 40–70% reduced risk of breast cancer in the BRCA 1/2 mutation carrier population, with effects particularly in premenopausal BRCA2 carriers, but more recently in both BRCA 1/2 [57,58].

#### 2.2.2. Salpingectomy with Delayed Oophorectomy

Emerging evidence in ovarian carcinogenesis research over the last several decades demonstrates convincing evidence for a tubal origin in HGSC which is associated with BRCA 1/2 mutation [59,60]. In addition, pre-menopausal RRBSO is associated with significant morbidity, including impact on quality of life, risk of osteoporosis, cardiovascular and metabolic disease, sexual functioning, and brain health [52]. Therefore, salpingectomy with delayed oophorectomy has been proposed as an alternative, ovary-sparing, risk-reducing intervention for HGSC in BRCA 1/2 mutation carriers. There exists high-quality evidence for improved quality of life with this option, regardless of eligibility for hormone replacement therapy, according to the preliminary TUBA trial, among others [61,62]. The extent of OC risk-reduction with this option is not yet known, and there are several large, ongoing non-randomized trials in the UK, the Netherlands, and the US [63,64,65]. At this time, the safety and efficacy of “partial” risk-reducing surgery for BRCA mutation carriers is preliminary at best, and the decision to undergo salpingectomy with delayed oophorectomy will depend on familial penetrance, fertility planning, and personal preference/risk tolerance [66,67]. While we await the results of the ongoing RCTs evaluating bilateral salpingectomy with delayed oophorectomy for HGSC prevention, it is relevant to note that a 2017 prospective study, looking at persistence of fimbrial tissue after salpingectomy followed by oophorectomy in the same operation, revealed a 10% (4/41) rate of persistent fimbrial tissue on the ovarian serosa [68]. This study is being repeated in BRCA1/2 carriers specifically at our dedicated familial ovarian cancer-reduction clinic to determine if the findings are reproduceable in a controlled surgical setting among carriers. 

#### 2.2.3. Concomitant Hysterectomy at RRBSO

Following decades of debate regarding elevated endometrial cancer (EC) risk in the BRCA 1/2 mutation carrier population, recent evidence largely supports an increased risk of EC in this group, particularly for uterine serous papillary cancer (USPC) [69,70,71,72,73]. Specifically, Nahshon et al. (2021) endorse an estimated 2-fold and 18-fold risk for EC and USPC, respectively [73]. For those who do not undergo risk-reducing hysterectomy, Shu et al. (2016) estimate a modeled lifetime risk of 3.5% of developing USPC and a 2% risk of dying from EC [69]. This risk is higher for BRCA 1 mutation carriers compared with BRCA 2 mutation carriers, with an estimated relative risk from a 2021 systematic review of 1.18 (95% CI, 0.7–2.0) for EC, and 1.39 (95% CI, 0.5–3.7) for UPSC specifically [71]. In addition, the literature reports a two- to seven-fold risk of EC with tamoxifen use, a selective estrogen receptor modulator commonly used to treat and prevent breast cancer [74]. This is particularly relevant to BRCA 1/2 mutation carriers who confer a higher risk of breast cancer themselves, and are offered tamoxifen as chemoprophylaxis or treatment for breast cancer, particularly in BRCA2 carriers. As such, concomitant hysterectomy at the time of RRBSO for EC prophylaxis has been considered [69].

Havrilesky et al. (2017) demonstrated that immediate hysterectomy at the time of RRBSO is cost-effective in the long term, and results in a mean life expectancy gain of 4.9 months [75]. In addition, hysterectomy at the time of RRBSO in pre-menopausal women eliminates the need for endometrial protection, thus negating the need for combination estrogen and progestin menopausal hormone therapy, which has implications for breast cancer risk compared to estrogen-alone therapy [76]. Furthermore, with the publication of the Women’s Health Initiative 20-year follow up data, one must consider that estrogen-alone hormone therapy with conjugated estrogens may actually be protective against breast cancer compared to placebo, not specifically in the BRCA 1/2 carrier population [77]. In contrast, hysterectomy increases the length of time, immediate cost, and resources required for this surgery, which has particularly challenging implications of expanded wait lists due to surgical delay since the pandemic began in 2020. Hysterectomy, even when performed laparoscopically, is reported to be associated with 3–4 times more major intraoperative complications, including intraoperative bleeding, urinary tract injury/infection, and bowel injury, when compared with adnexectomy alone [75,78]. In addition, the majority of EC presents early with the symptom of post-menopausal bleeding, and this cancer is therefore often diagnosed when the disease is still confined to the uterus, particularly for low-grade carcinoma [35]. At this time, hysterectomy at the time of RRBSO is not recommended in the Canadian and American guidelines for all carriers of BRCA1/2; however, individual risk factors for endometrial cancer, such as familial penetrance, Tamoxifen use, and obesity should be considered, as well as concomitant indications for hysterectomy, including, but not limited to, symptomatic fibroids and prolapse [54,56,71].

## 3. Conclusions

BRCA 1/2 mutation carriers face an increased risk of developing HGSC. A variety of pharmacologic and surgical interventions have been studied to determine their role in gynecologic malignancy prophylaxis in this population. Although the oral contraceptive pill is known to reduce the risk of ovarian cancer in the general and BRCA 1/2 mutated carrier population, its use for prophylaxis in this population is complicated by controversial data regarding an increased risk of breast cancer. Overall, breast cancer risk in carriers is not considered to be significantly elevated due to OCP use, and if also indicated for contraception, can serve as an effective OC prophylaxis in this population. Denosumab, ASA and NSAID therapy are being investigated for their role in risk-reduction for HGSC, but there is insufficient evidence at this time to recommend these therapies universally. With respect to surgical interventions, RRBSO remains the gold standard for HGSC prophylaxis in BRCA1/2 carriers. In the context of strong evidence supporting a tubal origin for BRCA-related ovarian cancer, salpingectomy with delayed oophorectomy is being considered as a menopause-sparing option. The risk-reduction quantification for this is unknown, but there is early strong evidence for improved quality of life, and clinical trials are underway globally. Finally, hysterectomy at the time of RRBSO is being considered for EC reduction; decision-making will need to be individualized based on EC cancer risk, tamoxifen use, and the need for HRT. At the multidisciplinary familial ovarian cancer center in Toronto, the above recommendations are followed. Each patient undergoes an individualized risk assessment and the appropriate risk-reduction options are offered, considering the patient’s goals, risk profile, and risk tolerance. 
